# Environmental risk factors of pregnancy outcomes: a summary of recent meta-analyses of epidemiological studies

**DOI:** 10.1186/1476-069X-12-6

**Published:** 2013-01-15

**Authors:** Mark J Nieuwenhuijsen, Payam Dadvand, James Grellier, David Martinez, Martine Vrijheid

**Affiliations:** 1 Centre for Research in Environmental Epidemiology (CREAL), Barcelona Biomedical Research Park, Dr. Aiguader 88, Barcelona, 08003, Spain; 2 Municipal Institute of Medical Research (IMIM-Hospital del Mar), Barcelona Biomedical Research Park, Dr. Aiguader 88, Barcelona, 08003, Spain; 3 CIBER Epidemiologia y Salud Pública (CIBERESP), Barcelona, Spain

**Keywords:** Meta-analysis, Pregnancy, Birth weight, Gestational age, Stillbirth, Congenital anomalies, Gestational age, Environmental exposures, Environmental tobacco smoke, Air pollution, Pesticides

## Abstract

**Background:**

Various epidemiological studies have suggested associations between environmental exposures and pregnancy outcomes. Some studies have tempted to combine information from various epidemiological studies using meta-analysis. We aimed to describe the methodologies used in these recent meta-analyses of environmental exposures and pregnancy outcomes. Furthermore, we aimed to report their main findings.

**Methods:**

We conducted a bibliographic search with relevant search terms. We obtained and evaluated 16 recent meta-analyses.

**Results:**

The number of studies included in each reported meta-analysis varied greatly, with the largest number of studies available for environmental tobacco smoke. Only a small number of the studies reported having followed meta-analysis guidelines or having used a quality rating system. Generally they tested for heterogeneity and publication bias. Publication bias did not occur frequently.

The meta-analyses found statistically significant negative associations between environmental tobacco smoke and stillbirth, birth weight and any congenital anomalies; PM_2.5_ and preterm birth; outdoor air pollution and some congenital anomalies; indoor air pollution from solid fuel use and stillbirth and birth weight; polychlorinated biphenyls (PCB) exposure and birth weight; disinfection by-products in water and stillbirth, small for gestational age and some congenital anomalies; occupational exposure to pesticides and solvents and some congenital anomalies; and agent orange and some congenital anomalies.

**Conclusions:**

The number of meta-analyses of environmental exposures and pregnancy outcomes is small and they vary in methodology. They reported statistically significant associations between environmental exposures such as environmental tobacco smoke, air pollution and chemicals and pregnancy outcomes.

## Background

Environmental exposures play an important role in the causation of disease. The developing foetus is thought to be particularly susceptible to environmental pollutants. Various epidemiological studies have suggested associations between environmental exposures such as air pollution, environmental tobacco smoke, pesticides, solvents, metals, radiation, water contaminants (disinfection by-products, arsenic, and nitrates) and chemicals (persistent organic pollutants (POPs), Bisphenol A, phthalates, and perfluorinated compounds (PFOS, PFOA)) and pregnancy outcomes such as pregnancy loss, stillbirth, fetal growth, preterm birth and congenital anomalies. These were described and evaluated recently in a number of reviews on environmental exposures and pregnancy outcomes [1-3]. Furthermore there have been a large number of (systematic) reviews on specific environmental exposures and pregnancy outcomes. In general the authors have suggested that while there is evidence supporting specific associations between environmental exposures and adverse pregnancy outcomes, evidence for other environmental exposures is limited. The latter may be partly due to the limited number of studies available, conflicting results from different studies, as well as the usual issues in epidemiological studies of bias and confounding, chance findings and limitations in exposure assessment.

One way to address some, but not all, of these issues is by combining information from various epidemiological studies and conducting a meta and/or pooled analyses to obtain overall summary estimates for an association between an environmental exposure and pregnancy outcome, and to evaluate any heterogeneity in the results. This may lead to a further insight into and/or better understanding of the association, improvement of methodology and, ultimately, to better risk management and policy making.

We aimed to describe the methodologies used in recent meta-analyses of environmental exposures and pregnancy outcomes. Furthermore, we aimed to report their main findings.

## Methods

A bibliographic search was carried out in December 2011 using MEDLINE (National Library of Medicine 2010). We limited our search to papers published in English and in the last 10 years. Initially we searched on “air pollution”, “environmental tobacco smoke”, “second hand smoke”, “persistent organic pollutants” (POPs), “PCB”, “pesticide”, “organic solvents”, “heavy metals”, “occupational exposure”, “radiation”, water contaminants such as “disinfection by-products”, “arsenic”, and “nitrates” and chemicals such as “Bisphenol A”, “phthalate”, and “PFOS PFOA” and “stillbirth”, “fetal growth”, “birth weight”, “preterm birth”, “gestational age” and “congenital anomalies” in PUBMED based on terminology used in recent reviews [1-3]. In this subset we viewed all the titles and abstracts and searched for the term “meta-analyses”. Furthermore we reviewed reports generated by the ENRIECO (Environmental Risks in European Birth Cohorts) project (http://www.enrieco.org). We only included studies that conducted meta-analyses to obtain summary estimates and evaluated heterogeneity between different studies. We did not include spontaneous abortion/miscarriage in the evaluation.

We reviewed each meta-analysis according to: the databases they used, whether meta-analysis guidelines were used (Meta-analysis Of Observational Studies in Epidemiology (MOOSE) or [4] Quality of Reporting of Meta-analyses (Quorom) statement, 2009 [5,6]), whether included studies were rated on quality (e.g. Newcastle-Ottawa scale [7] or Cochrane Handbook guidelines [8]), the statistics used to test for heterogeneity in the data (Cochran’s Q [9] or I^2^[10]), whether fixed [11] or random effects models [12] were used in the pooling of individual studies, and which tests of publication bias were used (funnel plots [13], Egger’s test [14], or Begg’s test [15]). Furthermore we checked whether sensitivity analyses had been carried out e.g. for influential studies by leaving one study out at the time, or analyses defined by subgroups.

## Results

### Bibliographic search

**Figure 1:**
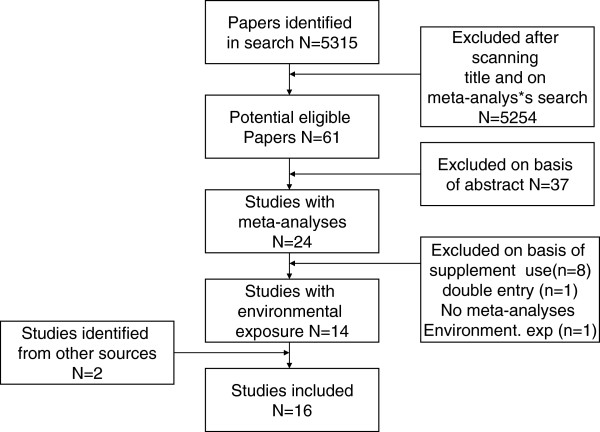
Flow diagram included and excluded studies.

In total we identified 5,315 papers in our search (Figure 1). After scanning the titles and conducting a further search for “meta-analyses”, we found 61 potentially eligible papers. We excluded 37 papers after reviewing the abstract because no meta-analysis was actually conducted, eight because the meta-analyses were for dietary supplement use, one because of double entry, and one after reading the paper and established that it contained no meta-analysis results on environmental exposures. Furthermore, we found two papers with meta-analyses through other sources [16,17]. Sixteen papers remained for detailed review (Table 1).

**Table 1:** Characteristics and methods used in the evaluated meta-analysis papers

**Study**	**N studies included**	**N subjects**	**Databases**	**Followed guidelines MOOSE**	**Quality rating**	**Cochran Q**	**I2**	**Random/fixed**	**Funnel plot**	**Egger test**	**Blegg’s**	**Sensitity analyses**
**Environmental tobacco smoke**												
Leonardi-Bee et al. 2008 [18]	58		MEDLINE, EMBASE, CINAHL, LILACS	yes	yes		yes	Random				yes
Salamasi et al. 2010 [19]	76	139 K	Medline, EMBASE, reference lists	yes	yes		yes	Random	yes			yes
Leonardi-Bee et al. 2011 [20]	19		MEDLINE, EMBASE	yes	yes		yes	Random	yes			yes
**Outdoor air pollution**												
Sapkota et al. 2010 [16]	20	Up to 1.9 M	ISI Web of Knowledge, PubMed			yes	yes	both		yes	yes	yes
Vrijheid et 2011 [21]	10	Up to 5.4 M	MEDLINE ISI Web of Science			yes		both		yes		yes
**Indoor air pollution**												
Pope et al. 2010 [22]	8/4	18 K/34 K	MEDLINE, EMBASE, Cochrane Controlled Trials Register Cumulative Index to Nursing and Allied Health Literaturee, Latin American and Caribbean Health Sciences Information System, System for Information on Grey Literature in Europe, Index to Conference Proceedings, PASCAL		yes	yes	yes	both	yes	yes	yes	yes
**Water contaminants**												
Hwang et al. 2008 [23]	6	Up to 3.3 M	PubMed			yes		both				yes
Nieuwenhuijsen et al. 2009 [24]	15	Up to 3.6 M	PubMed Review articles			yes		both	yes	yes		yes
Grellier et al. 2010 [25]	15	Up to 1.6 M	MEDLINE	yes	no	yes		both	yes	yes		yes
Nieuwenhuijsen et al. 2010 [17]	5		MEDLINE									
**POPs/PCB**												
Govarts et al. 2012 [26]	12	8 K	European birth cohorts http://www.enrieco.org			yes		both				yes
**Occupation**												
Logman et al. 2005 [27]	6	384 K	MEDLINE Toxline, Reprotox, EMBASE		yes	yes		both	yes	yes		
Romitti et al. 2007 [28]	7/5	3.5 K/64 K	MEDLINE					Random				yes
Rocheleau et al. 2009 [29]	9	376 K	PubMed					Random				yes
Pesticides												
Ngo et al. 2006 [30]	22	196 K	MEDLINE, EMBASE			yes	yes	both	yes	yes		yes
Ngo et al. 2010 [31]	7	135 K	MEDLINE, EMBASE		no	yes	yes	both	yes	yes		yes

The number of studies evaluated in the meta-analyses varied from 5 up to 76 (Table 1). The most used databases were MEDLINE/PUBMED and EMBASE. Only a minority reported following guidelines and using a quality rating system. Cochran’s Q was the most used test for testing for heterogeneity while almost half the studies used I^2^. Some studies reported using both. Half the studies reported using Funnel plots or the Egger test for evaluating publication bias, while only two used Begg’s test. All studies reported some form of sensitivity analyses. The topic with the most studies included was environmental tobacco smoke. A summary of results of the meta-analyses are given in Tables 2 and 3.

**Table 2:** Associations based on meta-analyses of air pollutants and birth outcomes

	**Still birth**	**Gestational age/pre term delivery**	**Birth weight /low birth weight/ small for gestational age**	**Congenital anomalies**
Environmental tobacco smoke	1.23 (1.09, 1.38) N=4 [18]	No stat sign association [19] No stat sign association [18]	Exposed vs. non exposed –60 g ( −80 g, -39 g) N=44 [19]-33 g (−16, -51) N=16 LBW 1.32, (1.07, 1.63) N=10 [20]	Exposed vs non exposed 1.18 (1.04, 1.34) N=12 [19] 1.13 (1.01, 1.26) N=7 [18]
Outdoor PM_10_		No stat sign association N=7 [16]	No stat sign association N=11 [16]	Atrial septal defects 1.14 (1.01, 1.28)/10 μg/m^3^ N=4 [21]
Outdoor PM_2.5_		PTB 1.15 (1.14, 1.16)/ 10 μg/m^3^ N=6 [16]	No stat sign association N=4 [16]	Too few studies
Outdoor NO_2_				Coarctation of the aorta 1.20 (1.00, 1.44)/10 ppb N=4 Tetralogy of Fallot 1.25 (1.02,1.51)/10 ppb N=4 [21]
Outdoor SO_2_				Coarctation of the aorta 1.04 (1.01, 1.08)/1 ppb N=4 Tetralogy of Fallot 1.04 (1.00, 1.08)/1 ppb N=4 [21]
Outdoor Ozone				No stat sign association [21]
Outdoor CO				No stat sign association [21]
Indoor air pollution from solid fuel use	1.51 (1.23, 1.85) Solid vs cleaner fuel N=4 [22]		Solid vs. cleaner fuel: -96.6 g (-68.5, -124.7) N=5 [22] LBW 1.38 (1.25, 1.52) N=8 [22]	

**Table 3:** Associations (95% CI) based on meta-analyses of contaminants and occupation and birth outcomes

	**Still birth**	**Gestational age/pre term delivery**	**Birth weight /low birth weight/ small for gestational age**	**Congenital anomalies**
Water contaminants-DBPs	high vs low 1.09 (1.02, 1.17) N=5 [17]	No stat sign association n=6 [23]	SGA: 1.01 (1.00, 1.02)/10 ug/L N=6 [23]	VSD 1.59 (1.21, 2.07) [25]Any congenital anomaly: high vs. low 1.17 (1.02, 1.34) N=5 high vs. low VSD 1.59 (1.21, 2.07) N=3 [24]
POPs-PCB153-DDE		No stat sign association N=12 [26] No stat sign association N=12 [26]	−150 g (−50, -250 g)/1 μg/L N=12 (Govarts et al. 2012)(26) No stat sign association N=12 [26]	
Occupation				Paternal solvent exposure Any malformation 1.47 (1.18, 1.83) N=6 Neural tube defects 1.86 (1.40, 2.46) N=5 Anencephaly 2.18 (1.52, 3.11) N=3 [27] Maternal pesticides Oral Clefts 1.37 (1.04, 1.81) N=5 [28] Maternal pesticides 1.36 (1.04, 1.77) N=7 [29] Paternal pesticides 1.19 (1.00, 1.41) N=8 [29]
Pesticides				Agent orange Birth defects 1.95 (1.59, 2.39) [30] Agent orange Spina Bifida 2.02 (1.48, 2.74) [31]

### Environmental tobacco smoke

Leonardi-Bee *et al.* conducted meta-analyses to determine the effects of environmental tobacco smoke (ETS) exposure on birth outcomes (birth weight and proportion of premature infants) [20]. Fifty eight studies were included; 53 used cohort design, 23 ascertaining ETS exposure prospectively and 30 retrospectively; and 5 used case–control design. In prospective studies, ETS exposure was associated with a 33 g (95% confidence interval (CI): 16, 51; I^2^=34%) reduction in mean birth weight, and in retrospective studies a 40 g (95% CI: 26, 54; I^2^=38.5%) reduction. ETS exposure was also associated with an increased risk of low birth weight (LBW, birth weight <2500 g; prospective studies: odds ratio (OR) 1.32, 95% CI: 1.07, 1.63; I^2^=54.7%); retrospective studies: OR: 1.22; 95% CI: 1.08, 1.37; I^2^=0%). The risk of small for gestational age (SGA, defined in the original studies as infant birth weight below the 10^th^ percentile for gestational age) was significantly associated with ETS exposure only in retrospective studies (OR: 1.21; 95% CI: 1.06, 1.37). There was no effect of ETS exposure on gestational age. They did not report on publication bias.

Salmasi *et al.* conducted extensive meta-analyses to determine whether there was an effect of ETS on pregnancy outcomes [19]. They only included studies comparing ETS-exposed pregnant women with those unexposed which adequately addressed active maternal smoking. Seventy-six studies were included with a total of 48,439 ETS exposed women and 90,918 unexposed women. Their primary outcome was perinatal mortality. The four main secondary outcomes were birth weight, gestational age at delivery, preterm birth (PTB) (< 37 weeks gestation), and LBW. Other secondary outcomes included were SGA (the 10th), intrauterine growth restriction (IUGR), congenital anomalies, stillbirth, and a number of others that we do not review here. ETS-exposed infants weighed less (−60 g; 95% CI: –80, –39 g) with a trend towards increased LBW (Relative risk (RR): 1.16; 95% CI: 0.99, 1.36; N=9), although the duration of gestation and preterm delivery were similar (0.02 weeks, 95% CI: –0.09, 0.12 weeks; n=17, and RR: 1.07; 95% CI: 0.93, 1.22; N=7). ETS-exposed infants had increased risks of congenital anomalies (OR: 1.17; 95% CI: 1.03, 1.34). The heterogeneity in the summary risk estimates of their outcomes ranged from an I^2^ test of 0–100%, and generally exceeded 75%, which is considered high. The heterogeneity was likely due to a variety of factors, including varying patient selection and the range of sample sizes. Further sensitivity analyses were carried out and these showed that in the analyses for birth weight, for example, infants born to mothers with self-reported ETS exposure had more heterogeneity (I^2^=100%) compared to those assessed biochemically (I^2^=54%). No further attempts were made to explore the heterogeneity. Except in the analysis for birth weight, funnel plots were relatively symmetrical, which suggests that publication bias was unlikely.

Leonardi-Bee *et al.* also conducted meta-analyses to determine the risk of adverse pregnancy outcomes due to ETS exposure in nonsmoking pregnant women [18]. The main outcome measures were spontaneous abortion, perinatal and neonatal death, stillbirth, and congenital anomalies. Nineteen studies were identified investigating these potential associations. ETS exposure significantly increased the risk of stillbirth (OR: 1.23, 95% CI: 1.09, 1.38; N=4; I^2^=0%) and congenital anomalies (OR: 1.13, 95% CI: 1.01, 1.26; N=7; I^2^=3%), although none of the associations with specific congenital abnormalities were individually significant. The number of studies included was generally small though. The degree of between-study heterogeneity was generally low (see above); publication bias results were not reported for stillbirth and congenital anomalies analyses.

### Outdoor air pollution

Sapkota *et al*. performed meta-analyses to quantify the association between maternal exposure to particulate matter with aerodynamic diameter 2.5 and 10 μm (PM_2.5_ and PM_10_) during pregnancy and the risk of LBW and PTB. They included 20 peer-reviewed articles providing quantitative estimate of exposure and outcome that met defined selection criteria [16]. They estimated a 15% increase in the risk of PTB for each 10- μg/m^3^ increase in PM_2.5_ (OR: 1.15; 95% CI: 1.14, 1.16), although with unlikely tight confidence intervals. The magnitude of risk associated with PM_10_ exposure was smaller (2% per 10-μg/m^3^ increase) and similar in size for both LBW and PTB, neither reaching formal statistical significance. They observed significant heterogeneity among studies that used PM_10_ as the exposure metric (LBW: I^2^=54%, p=0.01; PTB: I^2^= 73%, p<0.01), but not for studies that reported findings for PM_2.5_ (LBW, I^2^=57%, p=0.07; PTB: I^2^= 0.1%, p=0.42). They observed no significant publication bias, with p>0.05 based on both Begg’s and Egger’s bias tests.

Vrijheid *et al.* systematically reviewed epidemiologic studies on ambient air pollution and congenital anomalies and conducted meta-analyses for a number of air pollutant–anomaly combinations [21]. They identified 10 original epidemiologic studies. Meta-analyses were conducted if at least four studies published risk estimates for the same pollutant and anomaly group. Summary risk estimates were calculated for *a*) risk at high versus low exposure level in each study and *b*) risk per unit increase in continuous pollutant concentration. They conducted meta-analyses for 18 combinations of pollutants and cardiac anomaly groups and found that nitrogen dioxide (NO_2_) and sulphur dioxide (SO_2_) exposures were related to increases in the risk of coarctation of the aorta (OR per 10 ppb NO_2_: 1.20; 95% CI: 1.00, 1.44; OR per 1 ppb SO_2_: 1.04; 95% CI: 1.00, 1.08) and tetralogy of Fallot (OR per 10 ppb NO_2_: 1.25; 95% CI: 1.02, 1.51; OR per 1 ppb SO_2_: 1.04; 95% CI: 1.00, 1.08), and PM_10_ exposure was related to an increased risk of atrial septal defects (OR per 10 μg/m^3^: 1.14; 95% CI, 1.01, 1.28). Between study heterogeneity was identified (*p* < 0.10) in fewer than half of the analyses conducted, most consistently related to analyses of ventricular septal defects (VSDs). Egger test *p*-values were statistically significant for only 3 of the 68 meta-analyses they conducted, indicating that publication bias was unlikely.

### Indoor air pollution (solid fuel use)

Pope *et al.* conducted meta-analyses to quantify the relation of indoor air pollution from solid fuel use with birth weight and stillbirth [22]. They compared women using solid fuel with those using cleaner fuel. They found that solid fuel use was associated with increased risks of LBW (OR: 1.38; 95% CI: 1.25, 1.52) and stillbirth (OR: 1.51; 95% CI: 1.23, 1.85), and with reduced mean birth weight (-96.6 g; 95% CI: -68.5, -124.7). Heterogeneity was low (I^2^ = 0%) and there was no evidence for publication bias.

### Water contaminants-disinfection by-products

Hwang *et al*. conducted meta-analyses of chlorination by-products and birth defects [25]. They included six different studies from five publications and found an increased risk for VSD (OR: 1.59; 95% CI: 1.21, 2.07). They identified between-study heterogeneity for some congenital anomalies groups but did not test for publication bias.

Grellier *et al.* carried out a systematic review and meta-analysis of epidemiologic studies featuring original peer-reviewed data on the association of residential total trihalomethane (TTHM) exposure and health outcomes related to fetal growth and prematurity [23]. Fifteen studies were selected for the extraction of relative risks associating adverse birth outcomes to TTHM exposure. On a subset of eight studies, they found some evidence for an association between the third trimester TTHM exposure and SGA (OR: 1.01; 95%CI: 1.00, 1.02 per 10 μg/L TTHM). The Cochran test for homogeneity indicated a lack of heterogeneity among the studies, in contrast to a qualitative review of heterogeneity. The results of Egger’s regression test (both weighted and unweighted) demonstrated that the results appeared to be unaffected by publication bias, although low study numbers limited the robustness of this test. Similarly, funnel plots representing the results of such a low number of studies were considered hard to interpret.

Nieuwenhuijsen *et al.* conducted meta-analyses of disinfection by-products and stillbirth [17]. They found a summary OR of 1.09 (95% CI: 1.02, 1.17) when comparing the highest exposed group with the lowest exposed group. They did not report on heterogeneity and publication bias.

Nieuwenhuijsen *et al.* conducted meta-analyses for chlorination disinfection by-products (DBPs) and congenital anomalies [24]. They included 15 epidemiologic studies that evaluated a relationship between an index of DBP exposure (treatment, water source, DBP measurements, and both DBP measurements and personal characteristics) and risk of congenital anomalies. For all congenital anomalies combined, the meta-analysis gave a statistically significant excess risk for high versus low exposure to water chlorination or TTHM (OR: 1.17; 95% CI: 1.02, 1.34) based on a small number of studies. The meta-analysis also suggested a statistically significant excess risk for VSDs (OR: 1.58; 95% CI: 1.21–2.07), but this was based on only three studies, and there was little evidence of an exposure–response relationship. Four of the 17 analyses showed statistically significant heterogeneity. They found little evidence for publication bias, except for urinary tract defects and cleft lip and palate.

### POPs

Govarts *et al*. conducted meta-analyses of associations between POPs in maternal and cord blood and breast milk samples and gestational age and birth weight in 7,990 women enrolled in 15 study populations from 12 European birth cohorts between 1990 and 2008, which were part of the ENRIECO consortium (http://www.enrieco.org) [26]. Using identical variable definitions, they performed for each cohort linear regression of birth weight on cord serum concentrations of PCB 153 and p,p’-DDE while adjusting for gestational age and *a priori* selected covariates. The meta-analysis including all cohorts indicated a birth weight decrease of 150 g (95% CI: 50, 250 g) per 1 μg/L increase of PCB153, which was close to the range of exposure levels across the cohorts. They reported heterogeneity for the association between PCB153 and birth weight. No statistically significant association was found for DDE. They did not report on publication bias.

### Occupational exposure

Logman *et al.* conducted a meta-analysis to assess the risks of spontaneous abortions and major congenital anomalies following paternal exposure to organic solvents [27]. Six studies were included for major congenital anomalies, and they included quality scoring of the studies. Odds ratios were 1.47 (95% CI: 1.18, 1.83) for major congenital anomalies, 1.86 (95% CI: 1.40, 2.46) for any neural tube defect, 2.18 (95% CI: 1.52, 3.11) for anencephaly, and 1.59 (95% CI: 0.99, 2.56) for spina bifida. They did not find heterogeneity in the analyses. They did not report on publication bias.

Romitti *et al*. carried out meta-analyses to evaluate the risk of orofacial clefts associated with pesticide exposure [28]. Nineteen studies were included in the final analysis. For all phenotypes combined, maternal occupational pesticide exposure was associated with an increased risk of orofacial clefts (OR: 1.37; 95% CI: 1.04, 1.81). They reported that there was no statistically significant heterogeneity in the data but did not report on publication bias.

Rochelau *et al.* conducted meta-analyses of hypospadias associated with occupational maternal and parental exposure to pesticides [29]. Nine studies were included. Elevated but marginally significant risks of hypospadias were associated with maternal occupational exposure (RR: 1.36; 95% CI: 1.04, 1.77), and paternal occupational exposure (RR: 1.19; 95% CI: 1.00, 1.41). They found no heterogeneity in the reported risks by the studies. They found little evidence of publication bias.

### Pesticides

Ngo *et al*. conducted meta-analyses of studies looking at associations between the herbicide agent orange and congenital malformations [30]. They included 22 studies (205,102 subjects). The overall estimate of the RR of congenital anomalies in the Agent Orange exposed group as compared with the non-exposed group was 1.95 (95% CI: 1.59, 2.39). There was a significant variability across studies, with the heterogeneity Q statistic being 163 (P <0.001) and I^2^ of 0.87. The magnitude of association was higher in the Vietnamese population (RR: 3.0; 95% CI: 2.19, 4.12) than in non-Vietnamese veterans (RR: 1.29; 95% CI: 1.04, 1.59). In the Vietnamese studies, the magnitude of association was lower in cohort studies than in case–control studies. However, in non-Vietnamese populations, the association between Agent Orange and congenital anomalies was only found in cohort studies, not in case–control studies. In either cohort or case–control studies, significant heterogeneity of risk estimates was observed. I^2^ for all Vietnamese studies was 0.78 (P <0.001) and for the international veterans study was 0.85 (P < 0.001). They conducted sub-group meta-analyses stratified by intensity and duration of exposure. Funnel plots of all studies revealed a severely asymmetrical distribution, suggesting the presence of publication bias with the absence of small studies producing no statistically significant effects (Egger’s test: intercept = 3.75; P < 0.001). When studies were stratified by location of studies, the funnel plots and Egger’s test indicate the possibility of publication bias among Vietnamese studies (intercept = 3.06; P < 0.001) but not among non-Vietnamese studies (intercept = 3.13; P = 0.225). Moreover, the funnel plot and Egger’s test suggest some evidence of publication bias among all published studies (intercept = 3.80; P = 0.096).

Ngo *et al.* conducted meta-analyses of the herbicide agent orange and spina bifida [31]. Seven studies, encompassing two Vietnamese and five non-Vietnamese studies, were included. The overall RR for spina bifida associated with paternal exposure to agent orange was 2.02 (95% CI: 1.48, 2.74), with no statistical evidence of heterogeneity across studies. Non-Vietnamese studies showed a slightly higher summary RR (RR: 2.22; 95% CI: 1.38, 3.56) than Vietnamese studies (RR: 1.92; 95% CI: 1.29, 2.86). When analyzed separately, the overall association was statistically significant for the three case–control studies (OR: 2.25, 95% CI: 1.31, 3.86) and the cross sectional studies (RR: 1.97, 95% CI: 1.31, 2.96), but not for the three cohort studies (RR: 2.11; 95% CI: 0.78–5.73). Funnel plots revealed a symmetrical distribution with no evidence of publication bias (Egger’s test: intercept = 0.03; P = 0.96) for all studies including those not published, as well as for published studies only Egger’s test: intercept = 1.00, P = 0.6).

## Discussion

We have described the methodology used and main findings reported by meta-analyses of epidemiological studies investigating associations between environmental exposures and pregnancy outcomes conducted over the last 10 years and reported in the English language literature. In total we identified and described 16 meta-analyses meeting our inclusion criteria. The number of studies included in the reported meta-analyses varied greatly, with the largest number of studies available for environmental tobacco smoke. Only a small number of the studies reported to be following meta-analyses guidelines or using a quality rating system. Heterogeneity was reported in a number of the studies. Publication bias did not appear to occur frequently. The meta-analyses suggested statistically significant associations between ETS and stillbirth, birth weight and any congenital anomalies, PM_2.5_ and PTB, outdoor air pollution and possibly some congenital anomalies, indoor air pollution from solid fuel use and stillbirth and birth weight, PCB exposure and birth weight, disinfection by-products in water and stillbirth, SGA and possibly some congenital anomalies, occupational exposure to pesticides and solvents and some congenital anomalies, and agent orange and some congenital anomalies. However the number of studies included in the meta-analyses was often small, the exposure assessment limited and quality variable.

The relatively small number of meta-analyses (N=16) is at first glance perhaps surprising given the number of years of research in the area of environmental exposures and pregnancy outcomes. However as the meta-analyses showed, often there are not many studies with comparable data to conduct meta-analyses, except perhaps for ETS. Outcomes such as stillbirth and congenital anomalies studies are fairly rare and large numbers of subjects are needed, and for congenital anomalies the additional problem is case ascertainment and classification that can vary considerably between studies. Outcomes such as gestational age, birth weight, PTB, and LBW occur more frequently and are easier to study and compare among studies.

The main challenge to pooling studies using meta-analytical techniques is often thought to lie in the difficulties of combining studies with differences in exposure assessment, and therefore in obtaining comparable indices for meta-analyses. The ETS studies compared simple indices such as ETS exposed vs. non ETS exposed women [18-20] in the majority of studies retrospectively and to a great extent self-reported which may lead to exposure misclassification. However, in the (sensitivity) analyses there was little difference in the observed associations whether the data were obtained retrospectively or prospectively, or by self-report and/or some biochemical marker [19,20], which provides increased confidence in the results. Unfortunately there was little exploration of the importance of level and duration of the ETS exposure.

For outdoor air pollution, generally regulatory ambient measurements were used to derive exposure indices providing some numerical concentration values for the exposure response relationships. However there were considerable differences in terms of, for example, the temporal resolution of measurements or the distance of maternal home address to the measurements stations, which could lead to some doubt to how representative these were for the population.

The studies on disinfection by-products often used regulatory monitoring data of trihalomethanes in water, but generally did not include water intake measures or concentrations of other DBPs, which probably lead to exposure misclassification errors [17,23-25]. In some cases, analyses focused on high vs. low exposed groups which were not always directly comparable between studies.

The occupational exposure studies relied to a large extent on self reported job title and some assignment of exposure to the job title possibly leading to a considerable exposure misclassification [27-29]. Only Govarts *et al.* used biomonitoring data of POPs from different studies but had to use conversion factors to make comparable indices because POPs were measured in different media (Maternal blood, cord blood, and breast milk) [26]. Again this may increase measurement error. Furthermore they focused only on some specific POPs and not the whole POP mixture.

 In general, with various exceptions, non-differential measurement error/exposure misclassification may lead to attenuation in risk estimates and/or loss in power but could be compensated in the increased numbers of subjects in the combined studies [32]. A further option is to stratify analyses by the quality of the exposure assessment.

A further limitation of any meta-analysis of observational studies is residual confounding. Although the majority of individual studies had attempted to match or control for some important confounding variables such as maternal age, parity, socioeconomic status, alcohol, and drug use, the covariates included varied between studies. Since this may have resulted in residual confounding structures differing among the studies, it may have led to inappropriate pooling of heterogeneous study results in the meta-analysis. On the other hand, where studies with different underlying confounder structures show similar results, this will lead to increased confidence in the results.

Few studies reported having followed meta-analyses guidelines (MOOSE) or using a quality scoring system. Even though some did not report following guidelines, their approach appeared to be following the guidelines. One of the reasons for not following guidelines or using quality scores is probably the small number of studies included in general in the meta-analyses with the authors being familiar with the studies in the field. The few studies that included quality scores in their analysis did not see any difference in risk estimates between higher and lower quality studies [19,20].

The most used method to detect heterogeneity in the data was Cochran’s Q test. Only a small number of studies identified heterogeneity in their studies and this may be partly due to the fact that the tests for heterogeneity are not very powerful when the number of included studies is low [33,34]. If heterogeneity existed, generally no strategy was used in an attempt to reduce heterogeneity, for instance by making subgroups probably because of the small number of studies; however, some studies had already decided beforehand to conduct meta-analyses by subgroup (e.g. study design type). Salmasi *et al.* conducted meta-analyses overall and then stratified by the type of exposure assessment (self reported vs. biochemical) and thereby reduced the heterogeneity [19]. Sapkota *et al.* found less heterogeneity in studies of PM_2.5_ than PM_10_, suggesting that the former may be a better exposure index, since in PM_10_ may be acting as an imperfect surrogate for PM_2.5_ with differences between areas in how good to the surrogate is [16]. Of course, other explanations are also possible, including for example large variability in toxicity. At times a priori, or after testing even if there was no heterogeneity in the data, the meta-analyses used random effects models to take account of possible underlying difference between studies. This may have resulted at times in more conservative effect estimates (i.e. larger confidence intervals), but may better reflect the reality, where heterogeneity exist but may not be detected because of a small numbers of studies.

One issue to note is that authors often use I^2^ to estimate heterogeneity and we have referred to it as such here too. However I^2^ is not a measure of the magnitude of the between-study heterogeneity, nor a point estimate of between-study heterogeneity. It represents the approximate proportion of total variability in point estimates that can be attributed to heterogeneity [35]. The total variation depends importantly on the within-study precisions (essentially the sample sizes of the individual studies). Therefore, so must I^2^. Furthermore, I^2^ does not estimate a meaningful parameter, so should be regarded as a descriptive statistic rather than a point estimate [35]. Authors often omit to mention that the magnitude of heterogeneity can be quantified, using a point estimate of the among-study variance of true effects, often called τ^2^ (tau-squared). Thus, I^2^ may be viewed as the proportion of variability in the point estimates that is due to τ^2^ rather than within-study error [35]. A more appropriate descriptor for I^2^ would be a measure of inconsistency, since it depends on the extent of overlap in confidence intervals across studies.

Funnel plots and the Egger test were mostly used to detect publication bias. There was little publication bias observed. One of the reasons may be that many of the studies were time consuming and difficult to conduct and that therefore authors made great efforts to get the data published. Furthermore, a sufficient number of studies are needed to be able to detect publication bias, and where few studies are available, it may not be possible. Sensitivity analyses generally consisted of some subgroup analyses or leaving one study out at the time to determine if there were some influential studies. Generally the results did not change appreciably, suggesting that the results presented were robust.

## Conclusions

The number of meta-analyses of environmental exposures and pregnancy outcomes is small and they vary in methodology. Only a small number of the studies reported having followed meta-analysis guidelines or having used a quality rating system. However, they generally tested for heterogeneity and publication bias. Publication bias did not occur frequently. The available meta-analyses reported statistically significant associations between environmental exposures such as ETS, air pollution and chemicals and pregnancy outcomes like PTB, LBW, SGA, and congenital anomalies. We recommend future meta-analyses of the associations between environmental exposure and pregnancy outcomes to follow the available guidelines and report not only the combined effect estimates, but also the measures of heterogeneity, the method they use to account for heterogeneity (e.g. stratification of analyses or use of random effects models), and publication bias. The findings of these meta-analyses could provide a further insight into and/or better understanding of the association, improvement of methodology and, ultimately, to better risk management and policy making.

## Abbreviations

ETS: Environmental tobacco smoke; PM_2.5_: Particulate matter with cut off diameter <2.5 μm; PM_10_: Particulate matter with cut off diameter <10 μm; PCB: Polychlorinated biphenyls; PFOS: Perfluorooctane sulfonate; PFOA: Perfluorooctanoic acid; POPs: persistent organic pollutants; LBW: Low birth weight; PTB: Preterm birth; SGA: Small for gestational age; ppb: Parts per billion; VSD: Ventricular septal defect; TTHM: Total trihalomethanes; OR: Odds ratio; RR: Relative risk; 95%CI: 95% confidence interval.

## Competing interests

The authors declared no competing interest with respect to the authorship and/or publication of this article.

## Authors’ contributions

All authors contributed to the concept and design of the study, the review and revision of the article, and have approved the final version of the paper. MN carried out the database search and drafted the article.
